# Quantification and Impact of Cold Storage and Heat Exposure on Mass Rearing Program of *Bactrocera dorsalis* (Diptera:Tephritidae) Genetic Sexing Strain

**DOI:** 10.3390/insects11110821

**Published:** 2020-11-22

**Authors:** Jia Lin, Hanano Yamada, Ningfeng Lu, Guofu Ao, Weiwei Yuan, Xuxiang Liu, Pumo Cai, Minlin Zheng, Jianquan Yang, Qing’e Ji

**Affiliations:** 1 Institute of Beneficial Insects, Plant Protection College, Fujian Agriculture and Forestry University, Fuzhou 350002, China; Linjia@fafu.edu.cn (J.L.); 3190231027@fafu.edu.cn (N.L.); 2180203001@fafu.edu.cn (G.A.); 3190231047@fafu.edu.cn (W.Y.); 1180203015@fafu.edu.cn (X.L.); caipumo@fafu.edu.cn (P.C.); 000q030033@fafu.edu.cn (J.Y.); 2 Key Laboratory of Biopesticide and Chemical Biology, Ministry of Education, Fuzhou 350002, China; 3 Insect Pest Control Sub-Programme, Joint FAO/IAEA Programme of Nuclear Techniques in Food and Agriculture, A-1400 Vienna, Austria; H.Yamada@iaea.org; 4 Department of Horticulture, College of Tea and Food Science, Wuyi University, Wuyishan 354300, China

**Keywords:** mass-rearing, pupal cold storage, male quality, oriental fruit fly, short-term thermal exposure, fecundity

## Abstract

**Simple Summary:**

Storing insects at low temperature has been an effective approach for mass rearing programs, which can increase the shelf-life of bioagents and flexibly manipulate their pre-release periods. However, introducing insects to temperatures outside of their accustomed range can lead to stress and thereby influence their quality and competitiveness in the field. *Bactrocera dorsalis* is an economically important pest can devastate many fruits and vegetables, and cold storage has been used in storing, packing and shipping of sterile *B. dorsalis* males to control the wild population, but the impact of cold storage on the quality of *B. dorsalis* has never been investigated. Here, we examined the impacts of pupal cold storage on developmental parameters and quality of *B. dorsalis* adults. Overall, we found that pupal cold storage has a negative effect on some biological parameters of *B. dorsalis*, especially in fecundity. Thus, heat exposure was further conducted on the post-storage adults, and our results show that short-term heat exposure could partially compensate for the loss in fecundity in *B. dorsalis* adults resulting from the pupal cold storage. Our findings indicate that the flexibility of the *B. dorsalis* mass rearing program could be improved by utilizing cold storage and heat exposure.

**Abstract:**

Cold storage and heat exposure are crucial components of tephritid fruit fly mass-rearing programs, as they influence the development and fitness traits of produced flies. This work investigated the effects of cold storage on the pupal developmental parameters and quality of *Bactrocera dorsalis* (Hendel) genetic sexing strain (GSS) adults. Furthermore, the impact of short-term thermal exposure on the fecundity of *B. dorsalis* (GSS) that also underwent pupal cold storage was examined. Our results show that pupal development time, emergence rate, partial emergence rate, flight ability and fecundity were significantly affected by low temperature and pupal age and their interaction. Pupal cold storage did not pose negative impacts on the mating competition and response to methyl eugenol (ME) in the males. In addition, compared with the adults that were subjected to the same pupal storage protocol (five-day-old pupae stored at 13 °C), adult exposure to 41 °C for 1 h showed significant reparative effects on fecundity. In summary, the cold storage procedure of *B. dorsalis* (GSS) pupae has the potential to improve the flexibility and efficiency of mass-rearing schedules. Furthermore, short-term thermal exposure showed reparative effects on the fecundity costs induced by pupal cold storage in *B. dorsalis* (GSS).

## 1. Introduction

The oriental fruit fly, *Bactrocera dorsalis* (Hendel), is a highly polyphagous pest that can devastate a wide range of fruits and crops worldwide [1]. Organophosphate insecticides have been widely used to suppress the population of *B. dorsalis*; however, the resistance developed by the target pests and the adverse effects on non-target insects as well as mammals seriously limits this management strategy [2,3]. The sterile insect technique (SIT) has been regarded as an alternative tactic for the management of tephritid fruit flies. Based on the large-scale rearing of genetic sexing strains (GSS) that allows the efficient separation of sexes early in development, the SIT can disperse innumerable sterile males among the wild target population, resulting in infertile crosses and thus a continuous suppression of the tephritid fruit flies [4,5]. With the advantages of ecological safety and high species specificity, the SIT has successfully suppressed the populations of several important tephritid species [6,7,8]. However, some drawbacks related to the mass-rearing program have restricted the applicability and effectiveness of the SIT, including: (1) an insufficient production of target insects caused by disease infection and sub-optimal operations of the mass-rearing process [9], (2) a high cost and difficulty of rearing large numbers of sterile insects within a short period, (3) the release processes often being delayed or advanced because of severe weather, delayed transportation or unpredicted outbreaks of tephritid pests [10], and (4) the long-term, continuous mass-rearing of the fruit flies leading to genetic drift, inbreeding or genetic bottlenecks, which influence the genetic integrity of the GSS [11,12]. A method that could prolong the development duration of the sterile insects or archive the GSS in the laboratory could resolve or alleviate the problems involved in large scale rearing program of the plant pests, such as the insect cold storage technique [9].

Keeping insects at low temperature has been an effective approach for mass rearing programs to increase the shelf-life of bioagents, thereby ensuring a sufficient stockpile of insects, making release schedules more flexible and reducing the cost of maintaining insect colonies [13]. Considering these advantages, studies on insect cold storage were conducted as early as 85 years ago [14,15]. However, exposing insects to low temperature often led to chilling injuries and depleted energy reserves, which in turn influenced important fitness traits such as longevity, flight ability, mating success, fertility, and sperm production [16]. Therefore, more recent research has focused on optimizing the cold storage protocols to achieve the goal of storing, packing and shipping insects while keeping fitness costs at a minimum, and further improving the efficiency of large-scale production schedules [17,18]. In tephritids, several studies have been carried out to evaluate the effect of low temperature on the pupal development and the sublethal effects of extended cold storage on adult fitness traits of several economically important species to improve the flexibility and efficiency of the mass rearing programs. In terms of the effects of cold storage on the pupal development parameters, in the cases of *Bactrocera oleae* (Rossi) (Diptera: Tephritidae), *B. tryoni* (Froggatt) and *Anastrepha obliqua* (Macquart) (Diptera: Tephritidae), it has been shown that the extension in pupal development duration and the reduction in emergence rate were generally proportional to the decrease in storage temperature [19,20,21]. Previous work in our laboratory has shown that when three-day-old pupae of *B. dorsalis* (GSS) were stored at 13 °C and 16 °C for 10 or 15 days, the emergence rates were higher than 80% [22]. Regarding the quality cost of post-storage fruit flies, a study on *B. oleae* suggested that the oviposition ability of adults was adversely affected when pupae were stored at 11 °C [23]. Benelli et al. [21] indicated that the dry weight, lipid reserves and flight capacity of *B. tryoni* adults were significantly reduced as the pupal storing temperature decreased. For one-day-old pupae of *A. obliqua* developing at 18 °C and 20 °C, the flight ability was significantly reduced for both treatment temperatures compared to the control condition of 25 °C [20]. Nevertheless, comprehensive research related to the impact of pupal cold storage on the biological traits of *B. dorsalis* (GSS) adults is still absent.

Although cold storage is regarded as a crucial procedure to improve the rearing efficiency in large-scale programs, the adverse impacts on fecundity induced by low temperatures severely threatens the applicability of this technique [24]. Thus, means by which these detrimental effects could be repaired or minimized need to be considered. Recently, studies on *B. dorsalis* and *Zeugodacus cucurbitae* (Coquillett) (Diptera: Tephritidae) have shown that their fecundity increased after exposure to short-term heat treatments [25,26], and these results imply that this method could be applied to also improve the fecundity of fruit flies after pupal cold storage.

In this study, we aim to quantify the impacts of pupal cold storage on developmental parameters and quality of *B. dorsalis* (GSS) adults and to determine whether transient heat exposure can compensate for the adverse impacts on the fecundity of *B. dorsalis* (GSS) caused by pupal cold storage. For this purpose, we evaluated: (1) the effects of four “cold storage” temperatures (10, 13, 16 and 19 °C) on the emergence rate and development of *B. dorsalis* (GSS) pupae at three ages (1 d, 3 d, and 5 d old); (2) the flight ability, dry weight, mating competitiveness, response to methyl eugenol (ME) and fecundity of post-storage adults; and (3) the impacts of 1 h heat exposure on the mortality and fecundity of *B. dorsalis* (GSS) adults previously subjected to low temperatures during pupal stage.

## 2. Materials and Methods

### 2.1. Insects

The GSS of *B. dorsalis* was derived from a pupal colour sexing strain developed in Hawaii [27], and was transferred to the Institute of Beneficial Insects, Fujian Agricultural and Forestry University, Fuzhou, China, in 2007. The laboratory-reared *B. dorsalis* GSS female pupae were white, whereas the male pupae were brown [28]. The strain was then hybridized and backcrossed with wild Chinese *B. dorsalis* females [28]. The *B. dorsalis* (GSS) was reared under controlled conditions (25 ± 1 °C, 65 ± 5% RH and 12:12 (L:D) h photoperiod) and screened and filtered in each generation to ensure and maintain the stability of the sexing mechanism [29]. Adults were reared in a mesh cage (30 cm × 30 cm × 30 cm) and fed a diet of yeast extract:sugar (1:3), and a piece of wet cotton was used to provide water. The *B. dorsalis* larvae were reared with an artificial larval diet consisting of a mix of torula yeast, wheat bran, sugar, water, nipagin and sodium benzoate [30].

### 2.2. Experiment 1: Effect of Pupal Cold Storage on Developmental Parameters

The effects of two factors, cold temperature (10 °C, 13 °C, 16 °C, and 19 °C) and pupal age (1 d, 3 d or 5 d old), were assessed in terms of effects on developmental parameters. Here, one-day-old pupae developed at 25 °C for an additional 2 days or 4 days were considered the three-day-old and five-day-old pupae, respectively, and one-day-old pupae continuously developed at 25 °C were considered as the control group. One group of samples containing fifty brown pupae and fifty white pupae were transferred into a Petri dish (diameter: 9 cm). The dishes were wrapped with gauze to minimize the introduction of pathogens while ensuring ventilation. Five climatic incubators (PRX-25013, Safu, Ningbo, China) set at the specified temperature, 75 ± 5% RH and 0:24 (L:D) h photoperiod, were used to store the pupae. Emerged adults were then categorized by the following standards: (1) normal adult (fully emerged adult with normal morphology), (2) deformed adult (fully emerged adult with abnormal morphology, such as curly wings), (3) partially emerged (adult stuck in puparium until death), and (4) not emerged [21]. Normal adults and deformed adults were counted and collected from the dishes on the day of eclosion. Dishes were returned to climatic incubators after daily inspection. The emergence rate was calculated as ([*N* fully emerged adults + *N* deformed adult/*N* pupae]) × 100. Six replicates (i.e., dishes) of each treatment were conducted in this bioassay.

### 2.3. Experiment 2: Quality of Post-Cold Storage Adults

Pupae of different ages were stored under different constant temperature conditions until emergence, and emerged adults were used in this experiment.

#### 2.3.1. Dry Weight

Thirty males and thirty females emerged within 24 h of each treatment and controls were moved into 50 mL centrifuge tubes, respectively. These tubes were placed in a −20 °C freezer for 10 min to kill the adults, and were then transferred to an oven to be dried at 60 °C for 48 h. After the drying procedure, each dried fly was weighed individually using a semimicro balance (CP225D, accuracy of 0.01 mg, Sartorius, Göttingen, Germany).

#### 2.3.2. Flight Ability

One day before the first adult emerged, fifty brown pupae and fifty white pupae were placed in a 9 cm Petri dish placed at the base of a black cylindrical tube (inner diameter: 10 cm; height: 10 cm) in which the interior wall was daubed with talc powder to prevent adults from climbing out the tube. These tubes, including the dishes, were then placed in mesh cages and 30 W fluorescent lamps were positioned 20 cm above these cages, providing continuous light. This experiment was conducted at controlled conditions (25 ± 1 °C, 65 ± 5% RH). The fliers were collected into 10 mL centrifuge tubes every 12 h for 4 days to prevent them from returning to the black cylindrical tube. The flight ability was calculated as (*N* fliers/*N* pupae) × 100 [31]. For each treatment, six replicates (i.e., cages) were conducted.

#### 2.3.3. Fecundity

Ten treated females (15 days old) that had mated with the treated males were transferred into a mesh cage, provided with an oviposition plate with 10 holes containing a piece of filter paper soaked with 500 µL of orange juice. Females were permitted to oviposit over a period of 24 h. This test was performed at controlled conditions (25 ± 1 °C, 65 ± 5% RH and 12:12 (L:D) h photoperiod). After the oviposition trial, the total eggs in each replicate were counted under a stereomicroscope. Food and water were provided ad libitum during this experiment. For each treatment, six replicates (i.e., cages) were performed.

#### 2.3.4. Mating Competitiveness

After eclosion, adults from the different cold treatment groups were further separated by sex and placed into different cages. Control cages were also provided. One day before the bioassay, a cohort of 10 control males were exposed to 4 °C for 2 min to reduce their mobility and thereafter the thorax of control males were gently marked with pink fluorescent powder, and these marked males were then transferred to new cage [31]. Previous research has demonstrated that fluorescent powder marking did not influence male competitiveness [32]. Mating tests were performed by placing 20 treated males and 20 control males into a cage. Ten minutes after males acclimated to the territories, 20 virgin females were released into the cage. None of the adults used in this experiment had mated and all adults used were 15 days old. Experiments were performed from 18:00–22:00 and kept at 25 ± 1 °C, 65 ± 5% RH. All cages were visually inspected every 10 min, and mating pairs were collected gently with a 10 mL tube. Food and water were supplied in this bioassay. For each cold storage treatment, six replicates (i.e., cages) were used.

#### 2.3.5. Response to Methyl Eugenol (ME)

This experiment was performed in our laboratory’s experimental field site (119°13′58.5552″ E, 26°4′59.9412″ N). A potted guava tree (*Psidium guajava* L.; Myrtaceae) that served as a resting site for males was placed in the centre of the large field cages (1.2 m × 1.2 m × 1.5 m) [33], which were 10 m distanced from each other. To acclimate to the conditions, 100 mature males from the same cold storage treatment and 100 control mature males were simultaneously placed into the same cage 2 h before the bioassay. All adults used in this experiment were 15 days old. Control males were marked as described above (experiment 2: mating competitiveness). Cotton permeated with 50 µL ME was placed in the middle of the Tephritidae trap (Lvpusen, Quanzhou, China), and the trap was then hung in the guava tree with a wire. These trials were conducted starting at 8:00 am for 10 h, and the responding adults were counted and recorded under a ultraviolet light. Food and water were provided throughout the trial period. For each cold storage treatment, three replicates (i.e., cages) were performed.

### 2.4. Experiment 3: Impacts of Thermal Exposure to Post-Storage Adults of B. dorsalis (GSS)

Based on the results of the development period and emergence rate of *B. dorsalis* (GSS) pupae that underwent cold storage, it was found that storage of 5 d old pupae at 13 °C was a suitable protocol for preserving the genetic integrity of *B. dorsalis* (GSS) in the laboratory. Considering the adverse effects of cold storage and the potential reparative effect of thermal exposure on the fecundity of fruit flies, adults of this pupal storage treatment were used in the following experiments.

#### 2.4.1. Mortality after Thermal Exposure

Fifty females and fifty males that emerged within 24 h were transferred into a new cage, and then exposed to one of the following temperature treatments: 25 °C for 1 h (control), 33 °C for 1 h, 37 °C for 1 h, 41 °C for 1 h, and 45 °C for 1 h. Before the heat exposure, a cup containing 200 mL of water and a thermometer was placed into each climatic incubator to ensure that the temperature of water provided for adults was equal to the ambient temperature inside the climatic incubator. During the experiment, cotton soaked with 10 mL of water was supplied to the adults and replaced every 20 min. After heat exposure, all cages were returned to control conditions (25 ± 1 °C, 65 ± 5% RH and 12:12 (L:D) h photoperiod), and heat-shocked adults were collected into a Petri dish for recovery. One hour later, dead adults were counted. For each treatment, three replicates (i.e., cages) were conducted.

#### 2.4.2. Fecundity of *B. dorsalis* (GSS) after High-Temperature Exposure

The assessment of fecundity was performed as described above (experiment 2: fecundity). After the oviposition trial, the eggs of each replicate were counted, and 100 eggs of each replicate were carefully transferred to a new piece of filter paper placed on a 9 cm dish containing 30 g of larva diet. Subsequently, the dish was placed inside a plastic box that contained sand and was covered with gauze. All dishes were reared under control conditions (25 ± 1 °C, 65 ± 5% RH and 12:12 (L:D) h photoperiod). Sterile water was sprayed on the filter paper every 12 h to prevent the eggs from desiccating. The number of hatched eggs was inspected by using a stereomicroscope over a period of 4 days. The formula of hatchability was calculated as (the total number of eggs hatched per replicate/100 eggs used) × 100%. Pupae were collected daily from the sand by passing it through a sieve after day 6 of this experiment. The pupation rate was calculated as (the total number of pupae collected per replicate/100 eggs used) × 100%. For each treatment, six replicates (i.e., cages) were performed.

### 2.5. Statistical Analysis

Data analysis was performed using SPSS (SPSS Inc., Chicago, IL, USA). Data on deformed adult rate were arcsine square root-transformed, and dry weight data were square root-transformed to stabilize the variance prior to analysis [34]. Paired Student’s *t* test was used to analyse the significant differences in mating competitiveness and response to ME between the treatment and control groups. A univariate two-way ANOVA (Generalised Linear Model, GLM) was used to analyse the impact of the two independent factors (low temperature, pupal age) and their interaction on pupal developmental parameters and the fecundity and flight ability of post-storage adults. A univariate three-way ANOVA (GLM) was performed to analyse the effect of the three independent factors (low temperature, pupal age, and sex) and their interactions on dry weight. One-way ANOVA was used to analyse the impact of short-term heat exposure on the mortality and fecundity of post-storage adults. The differences between the treatments and control of experiment 1, experiment 2 and experiment 3 were assessed using a one-way ANOVA with Tukey’s honestly significant difference (HSD) test (*p* < 0.05) for multiple mean comparisons.

## 3. Results

### 3.1. Experiment 1: Effect of Pupal Cold Storage on Developmental Parameters

The development duration of pupae was significantly affected by temperature, pupal age, and the interaction of both (Table 1). Pupal development duration was progressively prolonged as temperature and pupal age decreased, and pupae developed at control temperature displayed a significantly shorter development time compared to the pupae stored at 13 °C (1 d, not emerged; 3 d, *p* < 0.001; 5 d, *p* < 0.001), 16 °C (1 d, *p* < 0.001; 3 d, *p* < 0.001; 5 d, *p* < 0.001) and 19 °C (1 d, *p* < 0.001; 3 d, *p* < 0.001; 5 d, *p* < 0.001), respectively (Table 2). Similarly, the emergence rate was also affected by temperature, pupal age, and their interaction (Table 1). However, contrary to the development time, emergence rate decreased as the storage temperature descended. Overall, compared to the control, a significant reduction in the emergence rate was observed in one-day-old and three-day-old pupae stored at 16 °C (*p* < 0.001, and *p* < 0.001, respectively) as well as in three-day-old and five-day-old pupae stored at 13 °C (*p* < 0.001, and *p* < 0.001, respectively) (Table 2). Furthermore, the effects of temperature and temperature × pupal age on the deformed adult rate were significant, while the effects of pupal age were not significant (Table 1). The lowest deformed adult rate (0.33%) was recorded for the control group, which was significantly lower than that of the one-day-old pupae stored at 16 °C (*p* < 0.01) and the five-day-old pupae stored at 13 °C (*p* < 0.001) (Table 2). The partial emergence rate was significantly affected by temperature, pupal age, and their interaction (Table 1). Increased storage temperature led to a reduction in partial emergence rates. Pupae developed at 13 °C resulted in a marked increase in partial emergence rates compared to control, with values of 42.67 ± 4.18 for three-day-old pupae (*p* < 0.001) and 55.50 ± 1.41 for five-day-old pupae (*p* < 0.001), respectively (Table 2).

### 3.2. Experiment 2: The Quality of Post-Storage Adults

#### 3.2.1. Flight Ability

Storage temperature, pupal age and their interaction had significant impacts on the flight ability of *B. dorsalis* (GSS) (Table 3). Overall, the flight capacity of adults was reduced as the pupal age and temperature decreased, while the control group showed the greatest flight capacity, which is remarkably higher than that of the other treatments except for the five-day-old pupae stored at 19 °C (*p* = 0.059) (Table 4).

#### 3.2.2. Dry Weight

Storage temperature and the age of the pupae had significant effects on the dry weight of resulting adults, while no significant effect of sex was observed (Table 3). Control adults presented significantly greater dry weights than the adults emerged from the three-day-old pupae (females, *p* < 0.001; males, *p* < 0.001) and five-day-old pupae (females, *p* < 0.05; males, *p* < 0.05) that were stored at 13 °C, while no significant differences were observed in the other treatments. Furthermore, the dry weight of adults from both sexes was reduced with the decrease in temperature and there were no differences between males and females in all treatments (Table 4).

#### 3.2.3. Fecundity

**Figure 1:**
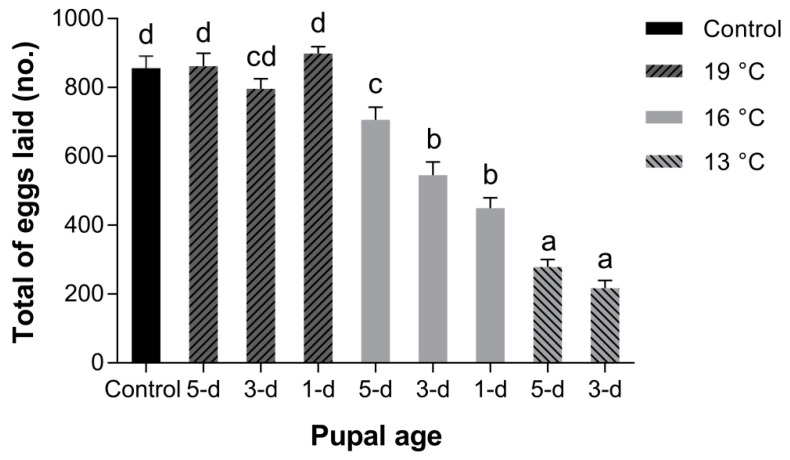
Fecundity of *B. dorsalis* (GSS) after pupae (1 d old, 3 d old and 5 d old) stored at temperature of 13, 16 and 19 °C. The mean value followed by the same letter does not differ significantly (*p* > 0.05) according to Tukey’s HSD test (one-way ANOVA).

In terms of fecundity, it was significantly affected by temperature, pupal age, and their interaction (Table 3). Our results reveal that pupae developed at 19 °C (1 d, *p* = 0.987; 3 d, *p* = 0.898; 5 d, *p* = 1.000) had no influence on the fecundity of *B. dorsalis* (GSS); however, keeping pupae at 16℃ (1 d, *p* < 0.05; 3 d, *p* < 0.001; 5 d, *p* < 0.001) or 13 °C (1 d, *p* < 0.001; 3 d, *p* < 0.001; 5 d, *p* < 0.001) led to a significant reduction in the number of eggs laid (per ten females) compared to the control (Figure 1).

#### 3.2.4. Mating Competitiveness

**Figure 2:**
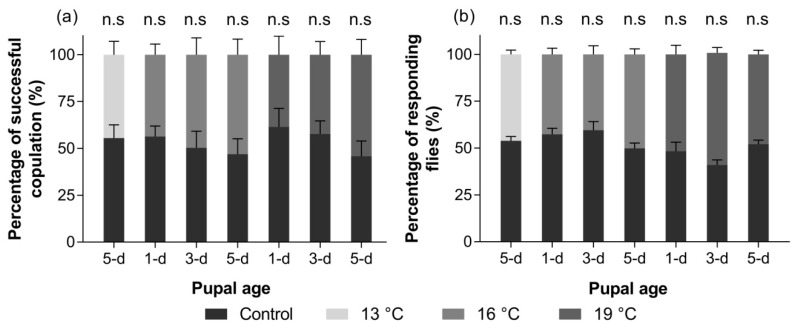
Mating competitiveness (mean ± SE) in the laboratory (**a**) and response to methyl eugenol (ME) in a field cage (**b**) of male *B. dorsalis* (GSS) after pupal cold storage.

Based on the analysis of the paired Student’s t test, male *B. dorsalis* (GSS) from all treatments did not show significant differences in mating competitiveness (Figure 2a).

#### 3.2.5. Response to ME

As shown by the results of the paired Student’s *t* test, there was no significant difference between each treatment and the control group (Figure 2b).

### 3.3. Experiment 3: Impact of Thermal Exposure to Post-Storage Adults of B. dorsalis (GSS)

#### 3.3.1. Mortality

**Figure 3:**
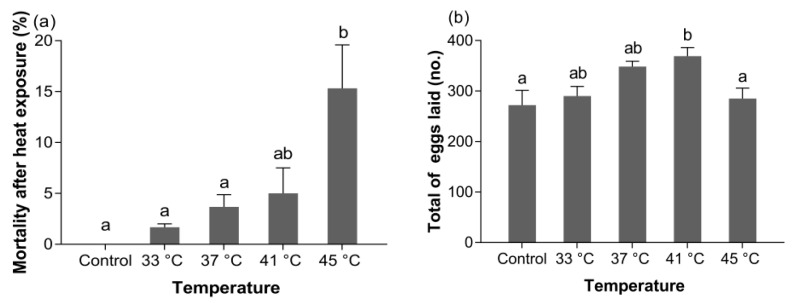

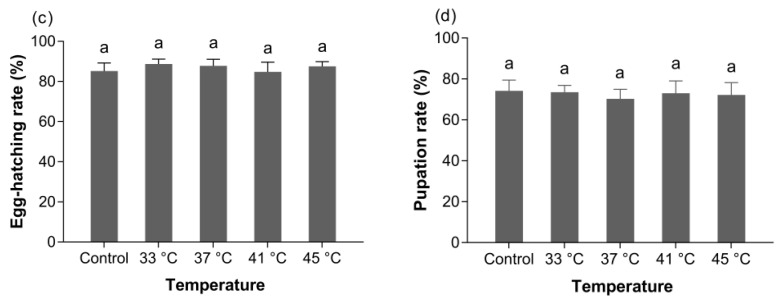
Impact of 1 h short-term heat exposure on the (**a**) mortality, (**b**) fecundity, (**c**) egg-hatching rate, and (**d**) pupation rate of post-storage adults (5 day-old pupae stored at 13 °C). The mean value followed by the same letter does not differ significantly (*p* > 0.05) according to Tukey’s HSD test (one-way ANOVA).

Transient thermal exposure had a significant effect on the mortality of *B. dorsalis* (GSS) (*F_4,14_* = 6.951, *p* < 0.01), and exposing newly emerged post-storage adults to 45 °C for 1 h led to a significant increase in mortality compared to the control (*p* < 0.01) (Figure 3a).

#### 3.3.2. Fecundity

Short-term heat exposure also resulted in significant effects on the number of eggs laid of *B. dorsalis* (GSS) (*F_4,29_* = 4.475, *p* < 0.05). In comparison with the control, the number of eggs laid by adults exposed to 41 °C for 1 h was significantly higher (*p* < 0.05) (Figure 3b). Furthermore, short-term heat exposure did not affect the egg-hatching rate (*F_4,29_* = 0.232, *p* > 0.05) (Figure 3c) or pupation rate (*F_4,29_* = 0.082, *p* > 0.05) (Figure 3d).

## 4. Discussion

This is the first study identifying the impacts of pupal cold storage on the quality of adults and determining the effects of heat exposure on the fecundity of post-storage *B. dorsalis* (GSS) adults. The results indicate that the developmental parameters and viability of pupae and the dry weight, flight ability and fecundity of post-storage *B. dorsalis* (GSS) adults were significantly affected by low temperature and pupal age; however, there were no differences observed between post-storage males and control males in their sexual competitiveness and response to ME. Furthermore, short-term high temperature exposure led to restorative effects on the fecundity of post-storage adults.

Low temperature can improve the convenience and efficiency of mass rearing programs through extending the shelf-life of insects and synchronizing their physiological characteristics, achieving the goal of increasing flexibility in the pre-release periods [35]. However, the delay of pupal developmental duration under low temperature exposure is usually coupled with the reduction in pupae viability [36,37]. Our results confirm that storing pupae at low temperature results in significant effects on the development time and emergence rate of *B. dorsalis* (GSS) pupae. The development time of pupae (one-day-old, and three-day-old) stored at 16 °C was prolonged to 21.22 days and 20.00 days, respectively, which was approximately twice as long as that of the control, and additionally, a decrease in emergence rate was observed. However, five-day-old pupae stored at 16 °C showed an extended pupal development time to 18.56 days with no adverse effects on the emergence rate. A low temperature of 13 °C negatively impacted the emergence rate of pupae (no adults emerged from the one-day-old pupae group), with values of 2.83% for three-day-old pupae and 15.00% for five-day-old pupae, even though the development time was quadruple and triple that of the control, respectively. Overall, there was a gradual reduction in adult emergence and a substantial increase in pupal development time as temperature and pupal age decreased. These results are consistent with previous studies on *B. dorsalis* (one-day-old pupae), which found that developmental delay increased and adult emergence declined as temperature decreased, but these effects occurred after treatments at a temperature of 16 °C and above [38,39].

The extent of reduced pupal survivorship is primarily based on the temperature and duration of storage [13], although we suggest that the pupal age used for storage is also a critical factor. In our study, the five-day-old pupae group showed better success in emergence rates compared to younger pupae stored at the same temperature. Similarly, in a pupal cold storage study on *Aphdius gifuensis* (Ashmead) (Hymenoptera: Aphidiidea), the emergence rate of three-day-old pupae was higher than that of the one-day-old and two-day-old pupae when stored at 5 °C [40]. The same results were observed for *Encarsia formosa* (Gahan) (Hymenoptera: Aphelinidae), in which the middle stage of pupae appeared to be associated with a lower mortality rate due to cold storage compared with the early stage of pupae [41]. The reasons why five-day-old pupae of *B. dorsalis* (GSS) had higher survival rates than one-day and three-day old pupae may be due to the mechanism of coarctate pupae, which determined early pupal stage is the time when the metamorphosis has just occurred, and the larval tissues had just undergone histolysis inside of the puparium and were waiting for further reassembly into exarate pupae, this is a critical and fragile stage that is susceptible to low temperature exposure [42]. Furthermore, the enhanced development in physiological function and energy reserves when pupae were left in optimal temperature conditions for longer resulted in higher chill tolerance, because various physiological dysfunctions induced by cold injury and energy consumed during low-temperature storage are essential factors influencing the survival of pupae [43,44].

Introducing insects to temperatures outside of their accustomed range can lead to stress and thereby decrease their biological fitness parameters including competitiveness in the field [45]. Our results show that decreasing pupae storage temperatures to 13 °C for *B. dorsalis* (GSS) pupae resulted in a marked reduction in dry weight in both sexes compared to the control. Long-term cold storage results in the excessive consumption of energy reserves, which thus negatively impacts the weight of the insects [13]. Similar results were found in the *B. tryoni*, in which the dry weight was significantly decreased when pupae were developed at subambient temperatures [21].

The cold storage procedure resulted in negative impacts on a crucial biological parameter of *B. dorsalis* (GSS), namely flight ability. In SIT programmes, the dispersibility of sterile tephritid primarily determines whether they can move to foraging, copulating and resting sites after being released in the field [31]. In our results, except for the five-day-old pupae developed at 19 °C, low temperatures led to a significant reduction in the flight ability of *B. dorsalis* (GSS) adults. Neuro-muscular dysfunctions together with the constant consumption of nutrients under low temperature conditions could explain the reduction in flight ability [13,46]. A study on *B. tryoni* found that the flight ability was strongly reduced after exposing pupae to cold temperatures of 19 °C and 17 °C [21]. Similarly, *A. obliqua* pupae stored at 18 °C and 20 °C showed lower flight ability than pupae developed at 25 °C [20].

The reproductive organs of insects are particularly vulnerable to low temperature effects [47], and the cost of fecundity is usually proportional to the chilling temperature and storage time [24,48]. Our results indicate that storage temperatures below 16 °C imposed negative effects on the fecundity of *B. dorsalis* (GSS). This is consistent with the study on *B. oleae*, which found that adults subjected to pupal cold storage made fewer oviposition holes on olives than the controls, and the number of progeny from the pupae developed from these olives were also fewer than the controls [23]. Research on *Trichogramma dendrolimi* (Matsumura) (Hymenoptera: Trichogrammatidae) has also shown that the lifetime reproduction of parasitoids after pupal cold storage was notably lower than that of control, regardless of the cold-storage treatment [49].

Mating competitiveness is one of the most crucial performance indicators of mass-reared sterile males as it is crucial for the successful application of the SIT as sterile males need to outcompete wild males for successful mating with wild females [50]. The effects of various factors on the sexual competitiveness of *B. dorsalis* have been determined, such as irradiation, the use of probiotics and insecticides [51,52]. However, the impacts of cold storage during the pupal stage on sexual competitiveness of *B. dorsalis* is still unknown. Our results indicate that pupal cold storage had no negative impacts on the mating competitiveness of *B. dorsalis* (GSS) males. Similarly, storage of one-day-old pupae of *Ceratitis capitata* (Wiedemann) (Diptera: Tephritidae) at 16 °C did not have any adverse effects on mating competitiveness [17]. Furthermore, post-storage *B. dorsalis* (GSS) males did not show any differences in the sensitivity to ME compared to the control. ME is a parapheromone that is a powerful attractant for many tephritid flies, including *B. dorsalis*, and has been proven to improve the mating success and total body protein of *B. dorsalis* males after its ingestion [28,53]. These results indicate that by exposing *B. dorsalis* (GSS) pupae to low temperature, and then the adults to ME, high quality sterile males can be produced in the mass-rearing programme, increasing the program’s chances of success.

Colony maintenance is both expensive and time-consuming. Furthermore, situations such as genetic drift, disease, machine breakdown and other emergency situations (such as epidemics) impede the strain maintenance of fruit flies [9,54]. Therefore, finding an approach to preserve the genetic integrity of valuable strains and alleviate the operational cost of continuous colony maintenance is necessary. Our data showed that the development time of *B. dorsalis* (GSS) pupae (5 d old) developed at 13 °C could be prolonged to 32.70 days, which can greatly reduce the frequency of the rearing processes. However, the fecundity of females following this treatment showed a sharp decline. Therefore, to repair the adverse impact on the fecundity of post-storage *B. dorsalis* (GSS), short-term heat exposure was conducted, showing that fecundity could be partially restored after adults were exposed to a temperature of 41 °C for 1 h. Further investigations have indicated that short-term high-temperature exposure has a positive effect on the oviposition behaviour of *B. dorsalis* and *Z. cucurbitae* [25,26]. The reason why transitory heat treatments can improve the fecundity of *B. dorsalis* (GSS) after chilling storage may be attributed to the impact of vitellogenin (Vg). The production of Vg is a crucial factor influencing ovarian maturation [55]. A study on *Megachile rotundata* (Fabricius) (Hymenoptera: Megachilidae) found that a fluctuating thermal regime increased the expression of Vg in overwintering prepupae and provided a reparative effect to chilling injuries induced by exposure to low temperature conditions [56].

## 5. Conclusions

Low temperature and pupal age strongly influence the development parameters and biological traits of *B. dorsalis* (GSS), except for the capacity of sexual competitiveness and response to ME. Therefore, to adjust to the different actual requirements of the mass rearing scheme, it is recommended to increase flexibility by using low temperature treatments while maintaining an optimal balance between quality and extending storage time as required. Furthermore, short-term heat exposure could partially compensate for the loss in fecundity in *B. dorsalis* (GSS) resulting from the pupal cold storage. Further studies are needed to utilize the method of mark–release–recapture tests to investigate the field performance of post-storage *B. dorsalis* (GSS) males, and the reparative mechanisms of transient heat exposure leading to enhanced fecundity of post-storage *B. dorsalis* (GSS) need to be elucidated through molecular techniques.

## Figures and Tables

**Table 1:** Univariate analysis of effects of factors on development parameters and fitness traits (flight ability, fecundity, dry weight) of *B. dorsalis* (genetic sexing strains (GSS)).

Biological Parameters	Factors	df	F	*p*-Value
Pupal development time	A	3, 52	11,628.11	<0.001
	B	2, 52	864.99	<0.001
	A × B	3, 52	502.20	<0.001
Emergence rate (%)	A	3, 54	1344.47	<0.001
	B	2, 54	40.32	<0.001
	A × B	3, 54	15.21	<0.001
Deformed adult rate (%)	A	3, 54	14.50	<0.001
	B	2, 54	2.26	0.116
	A × B	3, 54	10.47	<0.001
Partial emergence rate (%)	A	3, 54	393.99	<0.001
	B	2, 54	3.59	<0.05
	A × B	3, 54	10.00	<0.001

A: storage temperature; B: pupal age.

**Table 2:** Mean ± SE of pupal development time, emergence rate, adult morphological normality rate, deformed adult rate and partial emergence rate of *B. dorsalis* (GSS) pupae (1 d old, 3 d old and 5 d old) stored at temperature of 10, 13, 16 and 19 °C.

Storage	Pupal Age (d)	Development Time (Days)	Emergence Rate (%)	Deformed Adult Rate (%)	Partial Emergence Rate (%)
Control	-	11.05 ± 0.02 ^a^	97.00 ± 0.68 ^a^	0.33 ± 0.21 ^a^	1.17 ± 0.31 ^a^
19 °C	1	15.40 ± 0.11 ^d^	95.50 ± 1.06 ^a^	1.33 ± 0.33 ^a^	1.17 ± 0.31 ^a^
	3	14.78 ± 0.02 ^c^	94.83 ± 1.30 ^a^	1.83 ± 0.48 ^ab^	2.00 ± 0.68 ^a^
	5	13.78 ± 0.02 ^b^	96.00 ± 0.58 ^a^	0.67 ± 0.21 ^a^	1.17 ± 0.31 ^a^
16 °C	1	21.22 ± 0.22 ^g^	77.33 ± 1.36 ^b^	4.33 ± 0.76 ^bc^	6.67 ± 0.80 ^a^
	3	20.00 ± 0.21 ^f^	82.17 ± 1.80 ^b^	3.17 ± 0.60 ^ab^	5.00 ± 0.48 ^a^
	5	18.56 ± 0.09 ^e^	92.83 ± 0.95 ^a^	1.17 ± 0.48 ^a^	2.67 ± 0.95 ^a^
13 °C	1	-	-	-	-
	3	43.33 ± 0.24 i	5.17 ± 1.97 ^d^	2.33 ± 1.05 ^ab^	42.67 ± 4.18 ^b^
	5	32.70 ± 0.10 h	21.67 ± 2.29 ^c^	6.67 ± 1.05 ^c^	55.50 ± 1.41 ^c^
10 °C	1	-	-	-	-
	3	-	-	-	-
	5	-	-	-	-

The mean value in a column followed by the same letter does not differ significantly (*P* > 0.05) according to Tukey’s HSD test (one-way ANOVA). “-” means no adults emerged, and data of treatments without adults’ emergence were not conducted in the analyses.

**Table 3:** Univariate analysis of effects of factors on fitness traits (flight ability, fecundity, dry weight) of *B. dorsalis* (GSS).

Biological Parameters	Factors	df	F	*p*-Value
Flight ability (%)	A	3, 54	256.64	<0.001
	B	2, 54	43.95	<0.001
	A × B	3, 54	9.36	<0.001
Fecundity	A	3, 54	158.04	<0.001
	B	2, 54	9.29	<0.001
	A × B	3, 54	8.20	<0.001
Dry weight	A	3, 540	30.49	<0.001
	B	2, 540	3.76	0.024
	C	1, 540	1.12	0.273
	A × B	3, 540	1.98	0.115
	B × C	2, 540	0.68	0.507
	A × C	3, 540	0.28	0.844
	A × B × C	3, 540	0.56	0.637

A: storage temperature; B: pupal age; C: sex.

**Table 4:** Mean ± SE of flight ability and dry weight of *B. dorsalis* (GSS) of pupae (1 d old, 3 d old and 5 d old) stored at temperature of 13, 16 and 19 °C.

Storage Temperature	Pupal Age (d)	Flight Ability (%)	Dry Weight (mg)	
			Female	Male
Control	-	75.83 ± 2.41 ^a^	2.43 ± 0.04 ^Ac^	2.43 ± 0.06 ^Ac^
19 °C	1	50.50 ± 2.97 ^c^	2.32 ± 0.08 ^Abc^	2.36 ± 0.06 ^Abc^
	3	59.17 ± 2.69 ^bc^	2.39 ± 0.05 ^Abc^	2.41 ± 0.05 ^Ac^
	5	64.33 ± 3.21 ^ab^	2.42 ± 0.04 ^Ac^	2.44 ± 0.05 ^Ac^
16 °C	1	28.33 ± 3.47 ^d^	2.25 ± 0.05 ^Abc^	2.34 ± 0.05 ^Abc^
	3	38.50 ± 2.30 ^d^	2.26 ± 0.07 ^Abc^	2.38 ± 0.05 ^Abc^
	5	63.33 ± 2.30 ^b^	2.35 ± 0.05 ^Abc^	2.30 ± 0.05 ^Abc^
13 °C	1	-	-	-
	3	1.17 ± 0.48 ^e^	1.97 ± 0.06 ^Aa^	2.00 ± 0.05 ^Aa^
	5	4.83 ± 0.48 ^e^	2.14 ± 0.05 ^Aab^	2.16 ± 0.04 ^Aab^

The mean value in a column followed by the same lower letter does not differ significantly (*p* > 0.05) according to Tukey’s honestly significant difference (HSD) test (one-way ANOVA), and value of dry weight in a row followed by the same capital letter does not differ significantly (*p* > 0.05) according to paired *t* test.
